# MALDI-TOF MS Enables the Rapid Identification of the Major Molecular Types within the *Cryptococcus neoformans/C. gattii* Species Complex

**DOI:** 10.1371/journal.pone.0037566

**Published:** 2012-05-29

**Authors:** Carolina Firacative, Luciana Trilles, Wieland Meyer

**Affiliations:** 1 Molecular Mycology Research Laboratory, Centre for Infectious Diseases and Microbiology, Westmead Millennium Institute, Sydney Medical School–Westmead, The University of Sydney, Westmead Hospital, Sydney, Australia; 2 Grupo de Microbiología, Instituto Nacional de Salud, Bogotá, Colombia; 3 Laboratório de Micologia, Instituto de Pesquisa Clínica Evandro Chagas, Fundação Oswaldo Cruz, Rio de Janeiro, Brazil; Charité, Campus Benjamin Franklin, Germany

## Abstract

**Background:**

The *Cryptococcus neoformans*/*C. gattii* species complex comprises two sibling species that are divided into eight major molecular types, *C. neoformans* VNI to VNIV and *C. gattii* VGI to VGIV. These genotypes differ in host range, epidemiology, virulence, antifungal susceptibility and geographic distribution. The currently used phenotypic and molecular identification methods for the species/molecular types are time consuming and expensive. As Matrix-Assisted Laser Desorption Ionization-Time-of-Flight Mass Spectrometry (MALDI-TOF MS) offers an effective alternative for the rapid identification of microorganisms, the objective of this study was to examine its potential for the identification of *C. neoformans* and *C. gattii* strains at the intra- and inter-species level.

**Methodology:**

Protein extracts obtained via the formic acid extraction method of 164 *C. neoformans*/*C. gattii* isolates, including four inter-species hybrids, were studied.

**Results:**

The obtained mass spectra correctly identified 100% of all studied isolates, grouped each isolate according to the currently recognized species, *C. neoformans* and *C. gattii,* and detected potential hybrids. In addition, all isolates were clearly separated according to their major molecular type, generating greater spectral differences among the *C. neoformans* molecular types than the *C. gattii* molecular types, most likely reflecting a closer phylogenetic relationship between the latter. The number of colonies used and the incubation length did not affect the results. No spectra were obtained from intact yeast cells. An extended validated spectral library containing spectra of all eight major molecular types was established.

**Conclusions:**

MALDI-TOF MS is a rapid identification tool for the correct recognition of the two currently recognized human pathogenic *Cryptococcus* species and offers a simple method for the separation of the eight major molecular types and the detection of hybrid strains within this species complex in the clinical laboratory. The obtained mass spectra provide further evidence that the major molecular types warrant variety or even species status.

## Introduction

Cryptococcosis is a life-threatening infection caused for the inhalation of infectious propagules of the encapsulated yeasts *Cryptococcus neoformans* or *C. gattii* [1]. Cryptococcosis caused by *C. neoformans* var. *grubii* (serotype A), *C. neoformans* var. *neoformans* (serotype D) and the well-recognized hybrid AD is mainly an opportunistic infection of HIV patients [2]–[4], while *C. gattii* (serotypes B and C) is mainly affecting immunocompetent hosts, involving humans, domestic and wild animals [2], [5], [6]. Both species can be differentiated from each other by phenotypic methods [7], and at intra-specific level, by a range of genotyping methods, including PCR fingerprinting [8], [9], restriction fragment length polymorphism (RFLP) analysis [10], amplified fragment length polymorphism (AFLP) analysis [11], multilocus microsatellite typing (MLMT) [12] and multilocus sequence typing (MLST) [13], [14]. All currently used typing methods have identified eight major molecular patterns (VNI/AFLP1, VNII/AFLP1A for *C. neoformans* var. *grubii*, VNIII/AFLP3 for the AD hybrid, VNIV/AFLP2 for *C. neoformans* var. *neoformans* and VGI/AFLP4, VGII/AFLP6, VGIII/AFLP5 and VGIV/AFLP7 for *C. gattii*).

Strains of the eight major molecular types of the *C. neoformans*/*C. gattii* species complex differ also in their mating type distribution, virulence factors, disease characteristics, geographical distribution, epidemiology and ecological niche [1], [2], [15]. Consequently, the early and reliable diagnosis and the inter- and intra-specific identification of the agents causing cryptococcosis are of great importance for the clinicians, in order to allow for a prompt response and accurate treatment choice, especially in the case of emerging highly virulent genotypes, considering that there are differences in antifungal susceptibility patterns depending on the molecular type of the strain [6], [16]–[19]. However, correct identification of cryptococcal species and the molecular types by conventional methods can be sometimes difficult, expensive, and time consuming, as well as laborious preparation of media and reagents or extensive molecular investigations can be required [20], [21].

Recently as a substitute for phenotypic and genotypic methodologies used in clinical and applied laboratories, new, convenient and highly standardized technologies for the diagnosis and identification of infectious microorganisms to the subspecies level have been described. Among them, matrix-assisted laser desorption/ionization time-of-flight mass spectrometry (MALDI-TOF-MS) is being well renowned as an important tool for the rapid recognition of both bacteria [22], [23] and fungal agents, including the separation of the two pathogenic species *C. neoformans* and *C. gattii* [24]–[31]. This technique determines within a few minutes, specific patterns of peptides and protein mass spectra of either intact microbial cells or cellular extracts from pure cultures or from biological samples, allowing for the discrimination between species by comparison of the obtained spectrum with those included in a reference spectral library. Specific software packages can be used to perform the data acquisition, analysis, identification and result output [32], [33].

Therefore, the aim of this study was to evaluate the use and suitability of MALDI-TOF MS for a fast and reliable identification of strains of the two currently recognized pathogenic yeasts *C. neoformans* and *C. gattii* to the species/sub-species (major molecular types) level. This is the first time that this technology is used to identify and generate a library of the protein masses of clinical, environmental and veterinary isolates of the *C. neoformans*/*C. gattii* species complex representing all eight major molecular types previously determined by molecular methods.

## Results

### Strain Identification to the Currently Accepted Species Level

The analysis of 20 isolates for each of the eight major molecular types of the *C. neoformans*/*C. gattii* species complex by MALDI-TOF MS, resulted in a library of 960 individual mass spectra and 160 combined mass spectra, which allowed for the correct identification of all isolates according to the two currently recognized species *C. neoformans* and *C. gattii*, with identification scores ranging between 1.882 and 2.711 (Table 1). A 100% correct identification to the species level was obtained when the mass spectra generated were compared to the reference spectra of the five strains of *C. neoformans* and the two strains of *C. gattii* that are currently included in the MALDI Biotyper BDAL MSP library (2011) (see Materials and Methods). In general, the mass spectra obtained from *C. neoformans* were characterized by diverse signals in the region between 2050 and 9200 Da with strong peaks at approximately, 3340 and 6680 Da, while *C. gattii* spectra varied between 3030 and 9950 Da with strong peaks at approximately 3250, 4000 and 6650 Da. The peaks obtained at 7050, 7600 and 7900 Da, were found in both species. No isolate of the same species or molecular type produced identical spectra.

**Table 1:** Scores obtained for the identification of *Cryptococcus neoformans* and *C. gattii* isolates, using the original un-supplemented MALDI Biotyper BDAL MSP library (Bruker).

Species	Molecular type	Scores*
*Cryptococcus neoformans*	VNI	2.055–2.445
	VNII	2.319–2.358
	VNIII	2.135–2.161
	VNIV	2.083–2.204
*Cryptococcus gattii*	VGI	2.012–2.711
	VGII	2.055–2.166
	VGIII	2.055–2.500
	VGIV	1.882–1.945

* 2.300–3.000: highly probable species identification; 2.000–2.299: secure genus identification, probable species identification; 1.700–1.999: probable genus identification.

### Identification of the Major Molecular Types within the *C. neoformans/C. gattii* Species Complex

**Figure 1:**
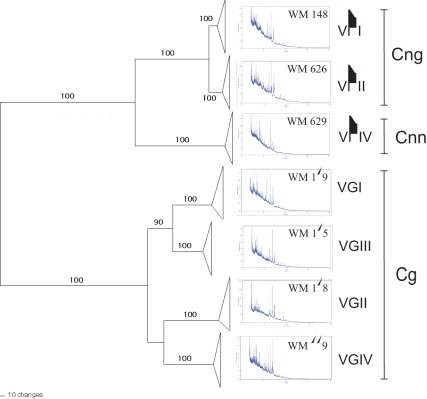
MLST phylogram of *Cryptococcus neoformans* and *C. gattii* isolates obtained using the ISHAM MLST consensus scheme for the *C. neoformans/C. gattii* species complex (*CAP59*, *GPD1*, *LAC1*, *PLB1*, *SOD1*, *URA5*, and IGS1), and associated mass spectra from the respective reference strains for each major molecular type (for strain info see Material and Methods); *Cnn*  =  *C. neoformans* var. *neoformans*, *Cng*  =  *C. neoformans* var. *grubii* and *Cg*  =  *C. gattii*; bootstrap values given above the lines.

**Figure 2:**
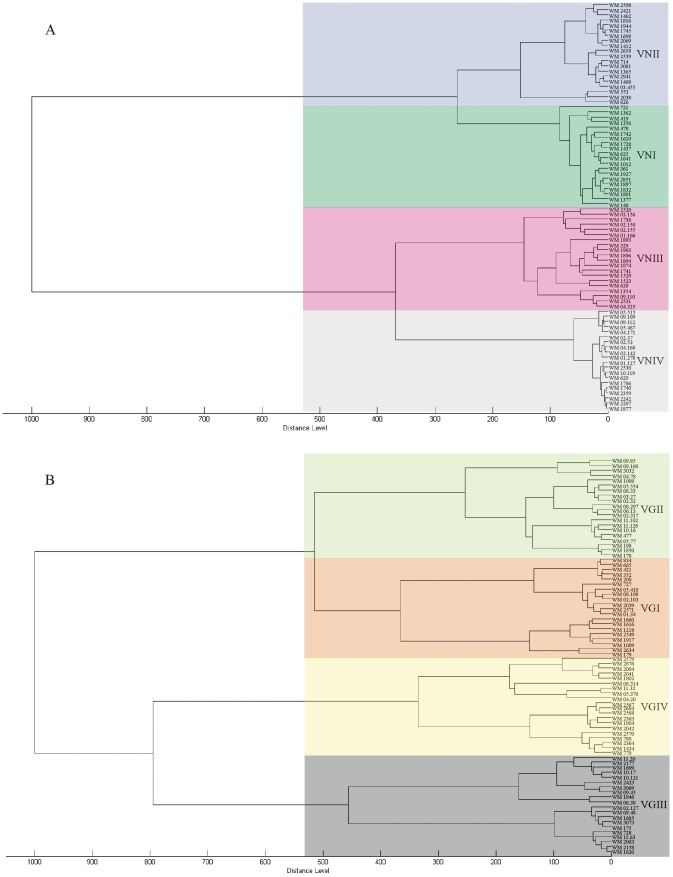
MSP dendrograms grouping A) mass spectra of *Cryptococcus neoformans* and B) *C. gattii* strains according to their major molecular type.

**Figure 3:**
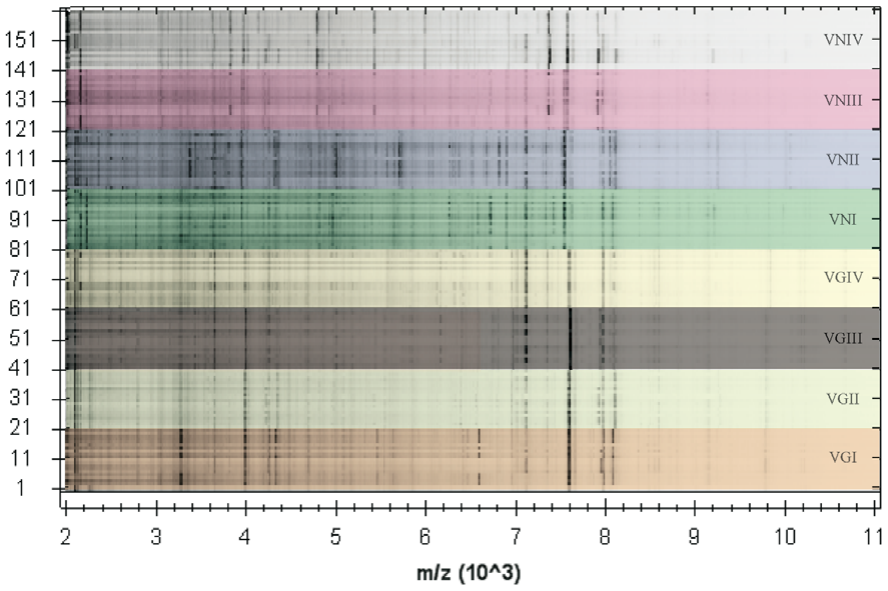
Gel view of the MSP of the obtained mass spectra of *Cryptococcus neoformans* and *C. gattii* strains according to their major molecular type.

In addition to the species identification, MALDI-TOF MS grouped the obtained mass spectra according to the eight major molecular types within the *C. neoformans/C. gattii* species complex. Representative spectra obtained from the reference strains for the seven haploid major molecular types are presented along with a phylogram generated from randomly selected strains of those major molecular types based on the concatenated sequences of the ISHAM MLST consensus loci (*CAP59*, *GPD1*, *LAC1*, *PLB1*, *SOD1*, *URA5*, and IGS1) [13] (Figure 1). The dendrograms obtained from the mass spectra shown in Figure 2A and 2B group the isolates according to the major molecular types VNI to VNIV for *C. neoformans* and VGI to VGIV for *C. gattii*, respectively. The isolates belonging to each molecular type produced distinct mass spectral signatures that were distinguishable from each other (Figure 1 and 3). The specific protein mass and the intensity of the obtained peaks characterizing each group of isolates within the same molecular type are clearly visible using the gel view option of the MALDI Biotyper software 3.0 (Bruker Daltonik GmbH) (Figure 3).

### Detection of Hybrids

MALFI-TOF MS resulted in a 100% correct identification of all studied AD hybrid strains (Figure 2 and 3). When analyzing four inter-species hybrid strains, previously identified by *URA5* RFLP, no clear correlation with any of the eight major molecular types was obtained. All hybrid strains were identified as belonging to the *C. neoformans/C. gattii* species complex with identification scores ranging between 1.728 and 2.304. The dendrogram generated with the mass spectra of the isolates, located the hybrids separately from the main clusters of the eight major molecular types. However, all the hybrid isolates grouped very close to at least one of the potential parental molecular types as previously determine by *URA5* RFLP analysis and Luminex suspension array [34] (data not shown).

### Reproducibility of the Obtained Spectra

Highly reproducible mass spectra were found when the proteins were extracted at two different occasions after the same time of incubation from the same isolate (data not shown). In addition, when analyzing the spectra of the proteins extracted from one or three colonies and incubated at 24 or 48 hours, no remarkable changes in the obtained spectra were found. The spectra obtained from one or three colonies or after 24 or 48 hours incubation presented peaks in the same mass range and intensity. No spectra were obtained when intact cells were used directly on the MALDI-TOF plate.

## Discussion

The distinction and characterization of pathogenic *C. neoformans* and *C. gattii* isolates at the species or subspecies level is important, mainly because of the differences in epidemiology, virulence, and antifungal drug susceptibility and subsequent disease outcome of the different strains [2], [6], [15], [16]–[19]. In this study, MALDI-TOF MS performed after a crude protein extraction generated highly informative mass spectra, which allowed for the accurate and reliable identification of 160 clinical, veterinary and environmental isolates of the two main pathogenic yeast species *C. neoformans* and *C. gattii*, as well as the detection of four newly recognized hybrid strains [34], in a shorter time (25 hours including culturing or less than one hour for crude protein extraction and MALDI-TOF analysis only), using less reagents and fewer experimental procedures, compared with conventional identification methods [8]–[14], [20], [21]. This is a fundamental improvement over the currently used molecular methods for the identification of the major molecular types within the *C. neoformans/C. gattii* species complex. *URA5* RFLP, which is currently the most commonly used method to determine the major molecular types, e.g. includes not only culturing the isolates, but also to carry out DNA extraction, *URA5* gene amplification and restriction enzyme digestion, followed by additional electrophoresis steps for the two latest procedures, with the whole process taking at least 3 days [10].

The herein obtained results are broadening the findings from recent studies, which reported that mass spectrometry-based identification proved to be a fast, reliable and sensitive alternative for the correct identification of a broad range of human pathogenic filamentous fungi and yeast [24]–[26], [30], including *C. neoformans* and *C. gattii*, although in previous studies, the isolates of both pathogenic *Cryptococcus* species were only identified to the species level [27]–[29], [31].

Additionally to the species differentiation among clinical *Cryptococcus* isolates, the MALDI-TOF technique also allowed for a clearly separation of the major molecular types of the *C. neoformans*/*C. gattii* species complex (Figure 1). These findings are in agreement with a previous study using MLST of the unlinked nuclear genes *ACT1*, *IDE*, *PLB1* and *URA5* [14], and with our MLST analysis (*CAP59*, *GPD1*, *LAC1*, *PLB1*, *SOD1*, *URA5*, and IGS1), in which separate or combined sequence analyses of all four or seven loci, respectively, revealed well-defined groups with significant support for each major molecular type, suggesting that the major molecular types are cryptic species and as such deserve either variety or species status [14], a fact that is reemphasized by the herein presented MALDI-TOF data. In our study, the spectra obtained from strains within each major molecular type were more homogeneous within *C. neoformans* than within *C. gattii*. In *C. neoformans*, the two varieties, *C. neoformans* var. *grubii* and *C. neoformans* var. *neoformans*, were clearly separated, which is in accordance with the grouping of the serotypes A and D, respectively [2], [15]. In *C. gattii,* despite the fact that four different genotypes have been well identified by several molecular methods, they have not yet been interpreted as distinct taxa [14], as both serotypes, B and C, occur in all genotypic groups [2], [15]. The obtained results most likely reflect a closer evolutionary relationship between the major molecular types of *C. gattii* (Figure 2A and 2B). *C. neoformans* var. *grubii* (VNI/VNII) separated from *C. neoformans* var. *neoformans* (VNIV) 24, VNI and VNII 4.7 and the *C. gattii* major molecular types VGIII and VGI 8.5, VGIV 11.7 and VGII 12.5 million years ago [14], [35].

MALDI-TOF MS proved once again its potential to be very useful for species differentiation, as has been already reported for the discrimination of e.g. *Mycobacterium* isolates, at both the species and strain level [22], for the identification of germ tube-negative *Candida* species [26] and for the identification of members of the *Pseudallescheria/Scedosporium* species complex [25]. For the first time, 20 mass spectra, composed of 6 single spectra for each major molecular type were added to the spectral library. The ability to correctly identify strains to the subspecies/major molecular types within the *C. neoformans/C. gattii* species complex was greatly enhanced through the addition of 160 new spectra to the MALDI Biotyper BDAL MSP library (2011), containing currently only five *C. neoformans* and two *C. gattii* spectra. From now on, it will be possible to use the mass spectra obtained for the recognition of the two currently recognized pathogenic *Cryptococcus* species, *C. neoformans* and *C. gattii,* and the differentiation of the major molecular types. This method can be used as a single identification technique or to complement traditional morphological or molecular identification/typing techniques like PCR fingerprinting, RFLP, AFLP, and MLST [7], [8]–[14].

As well as the identification of the currently recognized species and the major molecular types within the *C. neoformans/C. gattii* species complex, MALDI-TOF MS permitted the detection of recently characterized inter-species hybrid strains [34]. Until now intra- and inter-species hybrids have only been described on the basis of molecular analysis [2], [36]–[39]. The current study investigated one VNI/VGI and three VNI/VGII inter-species hybrids [34]. They were all correctly identified as belonging to the *C. neoformans/C. gattii* species complex but did not group within any of the eight major molecular types. In all cases they grouped closely to one of the parental major molecular type, VNI. It is currently unclear why they did not group in between both of the parental major molecular types. This could reflect different contribution of each parent strain to the gene composite of the hybrids or different expression levels of the proteins involved. A clear grouping of the well-known AD hybrid, VNIII/AFLP3 was obtained. Further studies with more proven hybrid strains for each molecular type combination are needed to assess the usability of the MALDI-TOF MS for the identification of hybrids and the parental origins.

Considering that different strains within the *C. neoformans*/*C. gattii* species complex are also characterized by different single nucleotide polymorphisms (SNPs) [13], [14] and that mass spectrometry based genotyping has recently been used for strain typing within several microorganisms, due to its advantages of speed and its ability to search for virulence factors and antibiotic resistance determinants [40], further studies are warranted to investigate the use of MALDI-TOF MS for cryptococcal strain typing.

In opposite to reports for other fungal species such as *Candida albicans*, for which it was reported that it is important to consider parameters that could affect mass signature reproducibility, like the number of cells used, the cell incubation conditions, the matrix compound used, or even the sample spotting technique [29], the number of colonies used in this study and the length of incubation of the strains or the spotting of the extract on the MALDI-TOF plate did not affect the obtained results. However, although protein purification steps were not required, it was always necessary to perform a protein extraction before placing the samples on the MALDI-TOF plate. Direct analyses of intact cells did not result in mass spectra, unlike for mass spectra analysis for bacterial identification [32] and for some other fungi, e.g. species of the genus *Candida* [33]. When analyzing *Cryptococcus* isolates, it is necessary to use ethanol in the protein extraction method, not only to avoid cellular aggregations, but also to inactivate this pathogen. Additionally, the alcohol fixation step has been proven to be critical to obtain high-quality mass spectra from yeast cells, and to be essential in the success of the technique for the identification of fungal species with the same accurate results as for bacterial species, for which no prior treatment or extraction method is needed [23], [29], [33]. The MALDI-TOF MS permitted as well to check for the reproducibility of the technique without using more reagents and almost at the same time, by spotting each sample extract six times on the MALDI-TOF target plate.

In conclusion, MALDI-TOF MS is an effective tool for the inter- and intra-specific differentiation of strains of the human pathogens *C. neoformans* and *C. gattii*. The comprehensive in-house library of the obtained mass spectra created in this study is substantially improving the MALDI Biotyper BDAL MSP library (2011), provided by Bruker, allowing for its use for the identification of the currently recognized two pathogenic *Cryptococcus* species. MALDI-TOF MS analysis provides for the first time a clear, rapid and highly reproducible method for the separation/identification of the major molecular types within the *C. neoformans/C. gattii* species complex and the detection of hybrids in the clinical diagnostic laboratory setting. Although the use of MALDI-TOF MS has until now been relatively limited for fungal identification compared with its application for bacterial identification, it is a cost-effective, rapid, sensitive and easy-to-handle tool for the early identification of these clinically important human pathogens. The diversity among the mass spectra obtained from isolates of the major molecular types, which is comparable with those obtained from other well defined yeast species, provides further evidence that the seven major haploid molecular types are cryptic species, which deserve separate species status.

## Materials and Methods

### Isolates

The usability of MALDI-TOF MS for the classification of the major molecular types within the *C. neoformans/C. gattii* species complex was investigated using 116 clinical, 32 environmental and 16 veterinary *C. neoformans* and *C. gattii* isolates (Table S1) [6], [8], [10], [11], [14], [34], [41]–[51]. All studied isolates were maintained at the culture collection of the Molecular Mycology Research Laboratory, University of Sydney at Westmead Hospital, Westmead, Australia. The references strains for *C. neoformans*, WM 148 (VNI, serotype A), WM 626(VNII, serotype A), WM 628 (VNIII, AD hybrid) and WM 629 (VNIV, serotype D) and for *C. gattii*, WM 179 (VGI, serotype B), WM 178 (VGII, serotype B), WM 175 (VGIII, serotype B) and WM 779 (VGIV, serotype C) were studied initially to generate reference spectra for each major molecular type. Subsequently, 20 isolates per molecular type were chosen randomly from over 3000 global cryptococcal strains to evaluate the ability of MALDI-TOF to distinguish between the eight major *C. neoformans*/*C. gattii* molecular types.

After analyzing all the spectra obtained from the 140 isolates with haploid genotypes (VNI, VNII, VNIV, VGI-VGIV) and 20 AD hybrids (VNIII), 4 additional inter-species hybrids strains were analyzed to assess the ability of the MALDI-TOF to detect potential hybrids. The strains tested included one VNI/VGI and three VNI/VGII hybrids previously identified by *URA5* RFLP analysis and Luminex suspension array [34] (Table S1).

### Major Molecular Types Determination

The major molecular type of all isolates was identified by RFLP analysis of the orotidine monophosphate pyrophosphorylase gene *(URA5*) via double digestion with the enzymes *Sau96I* and *HhaI* as previously reported [10].

### Multilocus Sequence Typing (MLST)

Nine strains of each of the seven haploid major molecular types of *C. neoformans* and *C. gattii* were studied by MLST using the ISHAM consensus MLST typing scheme for the *C. neoformans*/*C. gattii* species complex, which includes the following seven genetic loci: *CAP59*, *GPD1*, *LAC1*, *PLB1*, *SOD1*, *URA5*, and IGS1 [13]. The strains for the haploid *C. neoformans* major molecular type VNI were: WM 05.406, WM 05.473, WM 05.475, WM 05.479, WM 05.496, WM 05.553, WM 05.557, WM 09.170, WM 148, for VNII were: WM 05.483, WM 05.484, WM 05.485, WM 05.486, WM 05.490, WM 05.491, WM 05.523, WM 1816, WM 626 and for VNIV were: WM 01.126, WM 01.127, WM 02.142, WM 04.171, WM 04.172, WM 04.174, WM 05.469, WM 2242, WM 629. The strains for the haploid *C. gattii* major molecular type VGI were: WM 01.34, WM 02.103, WM 02.204, WM 05.410, WM 05.449, WM 08.108, WM 1009, WM 11.115, WM 179, for VGII were: WM 03.312, WM 03.697, WM 04.75, WM 04.78, WM 04.84, WM 05.272, WM 05.274, WM 05.275, WM 05.339, for VGIII were: WM 02.138, WM 02.139, WM 06.38, WM 09.43, WM 09.44, WM 09.45, WM 09.47, WM 11.10, WM 11.106 and for VGIV were: WM 04.20, WM 1220, WM 2363, WM 2579, WM 779, WM 780, WM 08.314, WM 1434, WM 2570.

A phylogram of the studied strains was obtained with PAUP* 4.0b10 (Figure 1). The obtained MLST data are incorporated into our in house *C. neoformans*/*C. gattii* databases accessible at mlst.mycologylab.org.

### MALDI-TOF MS

Isolates were cultured on Sabouraud dextrose agar and incubated for 48 h at 27°C. For the protein extraction one colony of each isolate was transferred separately into a 1.5 ml tube (Eppendorf) containing 300 µL of double-distilled water and mixed thoroughly for 1 minute. Subsequently, 900 µL of absolute ethanol was added in each tube and mixed for 1 minute. The samples were centrifuged for 2 minutes at 13000 rpm and the supernatant was removed. The pellet was dried at room temperature and according to the size of the pellet, 30 µL (small pellet) or 50 µL (big pellet) of formic acid (70%) was added. In addition, an equivalent volume of acetonitrile was added and the mix was centrifuge for 2 minutes at 13000 rpm. After cleaning the MALDI target plate, 1 µL of the supernatant was place in each well, dried at room temperature (5 minutes), and overlaid with 1 µL of the matrix containing a saturated solution of α-cyano-4-hydroxy-cinnamic acid in 50% acetonitrile/2.5% trifluoroacetic acid. The plate was let to dry at room temperature before placing in the MALDI-TOF reader. Each obtained extract was placed in 6 different spots on the plate to generate 6 combined mass spectra (MSP) per isolate. Mass spectra were generated using the MALDI Biotyper Automation Control software version 2.0.43.8 (BRUKER, Germany).

The proteins of the reference strains were extracted twice per isolate to assess the reproducibility of the results. To evaluate the effect of the number of colonies and the growth conditions of the isolates in the mass spectra obtained, one and three colonies of the reference strains incubated for 24 and 48 hours, were studied. Intact cells of the references strains were also placed directly in the MALDI-TOF plate in order to evaluate the extraction method.

### Data Analysis

The MALDI Biotyper software 3.0 (Bruker Daltonik GmbH) was used to identify the isolates, to visualize the mass spectra and to generate the MSP dendrograms. The scores for identification were expressed as log(score) values, indicating highly probable species identification (2.300–3.000), secure genus identification with probable species identification (2.000–2.299), and probable genus identification (1.700–1.999). The dendrograms were generated with the following settings: seuclidean as distance measure, ward linkage and 1 (one) as the maximum number of top levels nodes. One strain of each *C. flavus* (DSM70227T), *C. laurentii* (VML), *C. macerans* (DSM70822T), *C. albidus* (DSM 70197 PAH) and *C. uniguttulatus* (DSM 4652, DSM 70225), two of *C. gattii* (formerly classified as *C. bacillisporus* (RV490)) and five of *C. neoformans* (29 PSB, ATCC 14116 THL, CCM 8312, RV07 0218 and wVML) were used as reference spectra for the identification of the isolates, considering that they are the references for *Cryptococcus* species included in the All spectra generated were added to the in-house supplementary MALDI Biotyper BDAL MSP library (2011).

## Supporting Information

Table S1 ***Cryptococcus neoformans*** **and** ***C. gattii*** **isolates studied by matrix-assisted laser desorption/ionization time-of-flight mass spectrometry (MALDI-TOF-MS).**
(DOC)
