# Supplementary material for: MALDI-TOF MS Enables the Rapid Identification of the Major Molecular Types within the Cryptococcus neoformans/C. gattii Species Complex
Source: PLoS One. 2012 May 29;7(5):e37566. doi: 10.1371/journal.pone.0037566 (PMC3362595; doi:10.1371/journal.pone.0037566)
Supplement: Table S1 — Cryptococcus neoformans and C. gattii isolates studied by matrix-assisted laser desorption/ionization time-of-flight mass spectrometry (MALDI-TOF-MS). (DOC) [file pone.0037566.s001.doc]

**Table S1.** *Cryptococcus neoformans* and *C. gattii* isolates studied by matrix-assisted laser desorption/ionization time-of-flight mass spectrometry (MALDI-TOF-MS)

| **Type** | **WM number** | **Other collection number** | **Country** | **Source** | **Reference** |
| --- | --- | --- | --- | --- | --- |
| ***Cryptococcus neoformans var. grubii*** | | | | | |
| **VNI** | WM 1012 | 99-194-1904 | Brazil | Environmental | [10] |
|  | WM 1356 | TN/ENV/4 | India | Environmental | This study |
|  | WM 1362 | CN047 | New Zealand | Clinical | This study |
|  | WM 1377 | M-11 | Thailand | Clinical | This study |
|  | WM 1437 | UON 8705 | South Africa | Clinical | This study |
|  | WM 148 | CBS 10085, MH 57 (VNI standard) | Australia | Clinical | [10] |
|  | WM 1619 | NUAM 174 B | Mexico | Clinical | [10] |
|  | WM 1641 | NUAM 148 A, Mex-C26 | Mexico | Environmental | [10] |
|  | WM 1728 | HM 145535, Arg-C21 | Argentina | Clinical | [10] |
|  | WM 1742 | PMM CR-21, Chi-C9 | Chile | Clinical | [10] |
|  | WM 1801 | INDRE 5602, Mex-C2 | USA | Clinical | [10] |
|  | WM 1832 | BV 1742, Ven-C2 | Venezuela | Clinical | [10] |
|  | WM 1897 | IMIM 156 (C), 156 (C), Spa-E9 | Spain | Clinical | [10] |
|  | WM 1927 | H0058-I-21 | Colombia | Clinical | [10] |
|  | WM 2851 | UG-1647 | Uganda | Clinical | This study |
|  | WM 361 | CH 39-08/192 | Thailand | Clinical | This study |
|  | WM 419 | ATCC 90112 | USA | Clinical | [41] |
|  | WM 478 | HEC 11116 | Brazil | Clinical | [10] |
|  | WM 625 |  | Australia | Clinical | This study |
|  | WM 721 | TN/ENV/2 | India | Environmental | [14] |
| **VNII** | WM 03.455 | CN037 | New Zealand | Clinical | This study |
|  | WM 1365 | CN050 | New Zealand | Clinical | This study |
|  | WM 1408 | Hamden C3-1 | Brazil | Environmental | [11] |
|  | WM 1412 | RV 58146 | Zaire | Environmental | [42] |
|  | WM 1462 | UON 11536 | South Africa | Clinical | [14] |
|  | WM 1698 | HM 136105, Arg-C13 | Argentina | Clinical | [10] |
|  | WM 1745 | PMM CR-26, Chi-C12 | Chile | Clinical | [10] |
|  | WM 1816 | INDRE 5621, Mex-C17 | Mexico | Clinical | [10] |
|  | WM 1944 | H0058-I-43 | Colombia | Clinical | [10] |
|  | WM 2038 | H0058-I-626 | Colombia | Clinical | [10] |
|  | WM 2069 | H0058-I-902 | Colombia | Clinical | [10] |
|  | WM 2421 | CN041 | New Zealand | Clinical | This study |
|  | WM 2508 | Henley | Australia | Veterinary | This study |
|  | WM 2539 | TP 0915 | Australia | Clinical | This study |
|  | WM 2618 | MC-420(b) | India | Clinical | This study |
|  | WM 2941 | SA-2909 | South Africa | Clinical | This study |
|  | WM 3081 | Melville, W12 | Australia | Clinical | This study |
|  | WM 553 | HEC RJ-24, LMM 385 | Brazil | Environmental | [10] |
|  | WM 626 | W-20, CBS 10083 (VNII standard) | Australia | Clinical | [14] |
|  | WM 714 | Liimatta | Australia | Veterinary | This study |
| ***Cryptococcus neoformans var. neoformans*** | | | | | |
| **VNIV** | WM 01.127 | JEC 21 | USA | Clinical | [43] |
|  | WM 01.278 | A/D-11 | France | Clinical | This study |
|  | WM 02.142 | KRIMM 2 | Russia | Clinical | [14] |
|  | WM 02.37 | CDC R270 | Canada | Clinical | [6] |
|  | WM 02.52 | CDC R461 | Canada | Clinical | [6] |
|  | WM 04.168 | PMM CR-27, Chi-C13 | Chile | Clinical | [10] |
|  | WM 04.171 | CBS 7816 | Thailand | Environmental | [42] |
|  | WM 05.467 | LMM 547.18 | Brazil | Environmental | This study |
|  | WM 05.515 | 560000236, 405x, DE 8846 | Peru | Clinical | This study |
|  | WM 09.109 | CBS 9172 | Italy | Environmental | [44] |
|  | WM 09.112 | CBS 8710 | USA | Clinical | [45] |
|  | WM 10.119 | 2010:2:7B | Australia | Clinical | This study |
|  | WM 1706 | HM 137332, Arg-C36 | Argentina | Clinical | [10] |
|  | WM 1740 | PMM CR-12, Chi-C7 | Chile | Clinical | [10] |
|  | WM 1877 | IMIM 75 (c), Spa-E15 | Spain | Clinical | [10] |
|  | WM 2207 | TP 0416a, 1413 (B-3502) | USA | Environmental | This study |
|  | WM 2242 | TP 0415 alpha, B-3501, ATCC 52817 | USA | Clinical | [8] |
|  | WM 2359 | ATCC 52817, TP 0415 alpha, B-3501 | USA | Clinical | [8] |
|  | WM 2530 | JG-20 | USA | Clinical | This study |
|  | WM 629 | B 87455, CBS 10079 (VNIV standard) | Australia | Clinical | [10] |
| ***Cryptococcus gattii*** | | | | | |
| **VGI** | WM 01.34 | E 283 | Australia | Environmental | This study |
|  | WM 02.103 | Cr 10 | Argentina | Environmental | [10] |
|  | WM 05.410 | LMM 244 | Brazil | Clinical | This study |
|  | WM 08.108 |  | Australia | Veterinary | This study |
|  | WM 1009 | 99-194-1904 | Canada | Clinical | This study |
|  | WM 1218 | B33-19A | Australia | Environmental | This study |
|  | WM 1616 | NUAM 116, Mex-C1 | Mexico | Clinical | [10] |
|  | WM 1660 | UCLA 371 | USA | Clinical | [8] |
|  | WM 179 | CBS 10078, IFM 50893 (VGI standard) | Australia | Clinical | [10] |
|  | WM 1917 | IMIM 52 (A), Spa-E3 | Spain | Clinical | [10] |
|  | WM 200 | TCS-SC2 | Australia | Environmental | This study |
|  | WM 2039 | HOO58 I-628 | Colombia | Clinical | [10] |
|  | WM 2549 | KL-18, IMR C229/93A | Malaysia | Clinical | This study |
|  | WM 2571 | M27047, Q1169 | South Africa | Clinical | This study |
|  | WM 2643 | MC-S-34 | India | Clinical | This study |
|  | WM 352 | IDI 4A, IUM 96-2795, M 28966 | Italy | Environmental | This study |
|  | WM 421 |  | Papua New Guinea | Clinical | This study |
|  | WM 665 | E 268 | Australia | Environmental | This study |
|  | WM 727 | TP 0688, D1.12A, 22688 | USA | Environmental | [14] |
|  | WM 834 |  | Papua New Guinea | Clinical | This study |
| **VGII** | WM 02.317 | RB52 | Canada | Environmental | [6] |
|  | WM 02.32 | CDC R265 | Canada | Clinical | [6] |
|  | WM 03.27 | RAM 002 | Australia | Environmental | [46] |
|  | WM 04.78 | H0058-I-762 | Colombia | Clinical | [10] |
|  | WM 05.554 | LMM 1030 | Brazil | Clinical | This study |
|  | WM 05.77 | AV55, CBS 10090 | Greece | Clinical | [47] |
|  | WM 06.13 | CBS 7750, 451-SF13.2 | USA | Environmental | [6] |
|  | WM 06.33 | CBS 1930 | Aruba | Veterinary | This study |
|  | WM 08.297 | H0058-I-2792 | Colombia | Clinical | This study |
|  | WM 09.160 | N#24 | Australia | Veterinary | This study |
|  | WM 09.85 | 91/684, 91/183, 320, TP494, 13494 | Australia | Veterinary | This study |
|  | WM 10.16 | JS7, 10-00022 | USA | Veterinary | This study |
|  | WM 1008 | 99-194-1904 | Australia | Environmental | [46] |
|  | WM 11.102 | H0058-I-1449 | Colombia | Clinical | This study |
|  | WM 11.128 | JS 99 | USA | Veterinary | This study |
|  | WM 178 | CBS 10082, IFM50894, (VGII standard) | Australia | Clinical | [10] |
|  | WM 1850 | BV 1218, Ven-C20 | Venezuela | Clinical | [10] |
|  | WM 198 | VPB 571-015, McBride (M1) | Australia | Veterinary | [48] |
|  | WM 3032 | NT-14 | Australia | Clinical | [46] |
|  | WM 477 | HEC 11102, LMM 21 | Brazil | Clinical | [10] |
| **VGIII** | WM 02.127 | 2361 | Guatemala | Clinical | [10] |
|  | WM 06.38 | B4546 | USA | Clinical | [14] |
|  | WM 09.45 | 07-11763 | USA | Veterinary | This study |
|  | WM 09.48 | 08-6825 | USA | Veterinary | This study |
|  | WM 10.121 | 10-02-697 | USA | Veterinary | This study |
|  | WM 10.17 | 09-11987 | USA | Veterinary | This study |
|  | WM 11.20 | H0058-I-3306 | Colombia | Environmental | This study |
|  | WM 11.63 | PWQ 1098 | Australia | Clinical | This study |
|  | WM 1620 | NUAM 190, Mex-C5 | Mexico | Clinical | [10] |
|  | WM 1665 | UCLA 380C | USA | Clinical | [8] |
|  | WM 1699 | HM 136353, Arg-C05 | Paraguay | Clinical | [10] |
|  | WM 175 | 689, F 07, CBS 10081 (VGIII standard) | USA | Environmental | [10] |
|  | WM 1846 | BV 19, Ven-C16 | Venezuela | Clinical | [10] |
|  | WM 2063 | H0058-I-792 | Colombia | Clinical | [10] |
|  | WM 2158 | H0058-I-604 | Colombia | Environmental | [10] |
|  | WM 2177 | H0058-I-731 | Colombia | Environmental | [10] |
|  | WM 2423 | CN043 | New Zealand | Clinical | [46] |
|  | WM 3069 | H0058-I-859 | Colombia | Environmental | [10] |
|  | WM 3073 | H0058-I-964 | Colombia | Environmental | [10] |
|  | WM 728 | TP 0686, D1.10F, 22686 | USA | Environmental | [14] |
| **VGIV** | WM 04.20 | M27055, 25229 | South Africa | Clinical | [14] |
|  | WM 05.376 | H0058-I-1686 | Colombia | Environmental | This study |
|  | WM 08.314 | 16-1664 | Australia | Veterinary | This study |
|  | WM 11.32 | JS 81 | USA | Veterinary | This study |
|  | WM 1434 | UON 11135 | South Africa | Clinical | This study |
|  | WM 1802 | INDRE 5604, Mex-C3 | Mexico | Clinical | [10] |
|  | WM 1804 | INDRE 5606, Mex-C5 | Mexico | Clinical | [10] |
|  | WM 2004 | H0058-I-256 | Colombia | Clinical | [10] |
|  | WM 2041 | H0058-I-642 | Colombia | Clinical | [10] |
|  | WM 2042 | H0058-I-645 | Colombia | Clinical | [10] |
|  | WM 2363 | B-5742, M 30826 | India | Clinical | [14] |
|  | WM 2364 | B-5748, M 30827 | India | Clinical | [14] |
|  | WM 2567 | M27042, X242 | South Africa | Clinical | This study |
|  | WM 2568 | M27043, X303 | South Africa | Clinical | This study |
|  | WM 2570 | M27046, P2244 | South Africa | Clinical | This study |
|  | WM 2579 | M27056, P2238 | South Africa | Clinical | This study |
|  | WM 2604 | M31499, 4357 | South Africa | Clinical | This study |
|  | WM 2876 | V00869 | South Africa | Clinical | [14] |
|  | WM 779 | King Cheetah, CBS 10101, IFM 50896  (VGIV standard) | South Africa | Veterinary | [10] |
|  | WM 780 | V00709 | South Africa | Clinical | [14] |
| **Hybrids** | | | | | |
| **VNIII** | WM 01.166 | HC3 | Brazil | Clinical | [10] |
|  | WM 02.150 | KRIMM 10 | Russia | Clinical | [49] |
|  | WM 02.155 | KRIMM 15 | Russia | Clinical | [49] |
|  | WM 02.156 | KRIMM 16 | Russia | Clinical | [49] |
|  | WM 04.325 | 132448 | Australia | Clinical | This study |
|  | WM 09.110 | CBS 10512 | USA | Clinical | This study |
|  | WM 1354 | TBS 28 | India | Clinical | This study |
|  | WM 1523 | RKI-A208 | Germany | Environmental | [50] |
|  | WM 1529 | RKI-M364/98 | Germany | Clinical | [50] |
|  | WM 1738 | PMM CR-10, Chi-C5 | Chile | Clinical | [10] |
|  | WM 1741 | PMM CR-16, Chi-C8 | Chile | Clinical | [10] |
|  | WM 1874 | IMIM 4 (a), Spa-E19, LA192 | Spain | Environmental | [10] |
|  | WM 1893 | IMIM 1 (C), 1 (C), Spa-E10 | Spain | Clinical | [10] |
|  | WM 1894 | IMIM 2 (C), 2 (C), Spa-E11 | Spain | Clinical | [10] |
|  | WM 1896 | IMIM 155 (C), 155 (C), Spa-E8 | Spain | Clinical | [10] |
|  | WM 1903 | IMIM 154 (C), Spa-E7 | Spain | Clinical | [10] |
|  | WM 2520 | JG-10 | USA | Clinical | This study |
|  | WM 2531 | JG-21 | USA | Clinical | This study |
|  | WM 329 | IUM 90-7031 | USA | Clinical | This study |
|  | WM 628 | 88B5400, CBS 10080 (VNIII standard) | Australia | Clinical | [51] |
| **VNI/VGI** | WM 2617 | MC-420(a) | India | Clinical | [34] |
| **VNI/VGII** | WM 05.272 | H0058-I-1959 | Colombia | Clinical | [34] |
|  | WM 05.459 | LMM 558 | Brazil | Clinical | [34] |
|  | WM 05.532 | LMM 868 | Brazil | Clinical | [34] |
